# Interplay of TRIM2 E3 Ubiquitin Ligase and ALIX/ESCRT Complex: Control of Developmental Plasticity During Early Neurogenesis

**DOI:** 10.3390/cells9071734

**Published:** 2020-07-20

**Authors:** Ashwin Lokapally, Herbert Neuhaus, Juliane Herfurth, Thomas Hollemann

**Affiliations:** 1 Institute for Physiological Chemistry, Martin-Luther University Halle-Wittenberg, Hollystrasse 1, 06114 Halle, Germany; ashwinl@wi.mit.edu (A.L.); herbert.neuhaus@medizin.uni-halle.de (H.N.); juliane.herfurth@chemie.uni-halle.de (J.H.); 2 Whitehead Institute for Biomedical Research, 455 Main Street, Cambridge, MA 02142, USA

**Keywords:** Trim2, Alix, *Xenopus*, neural development, neural differentiation, apoptosis, ataxia

## Abstract

Tripartite motif 2 (TRIM2) drives neurite outgrowth and polarization, is involved in axon specification, and confers neuroprotective functions during rapid ischemia. The mechanisms controlling neuronal cell fate determination and differentiation are fundamental for neural development. Here, we show that in *Xenopus*, *trim2* knockdown affects primary neurogenesis and neural progenitor cell survival. Embryos also suffer from severe craniofacial malformation, a reduction in brain volume, and the loss of motor sensory function. Using a high-throughput LC-MS/MS approach with GST-Trim2 as bait, we pulled down ALG-2 interacting protein X (Alix) from *Xenopus* embryonic lysates. We demonstrate that the expression of *trim2*/TRIM2 and *alix*/ALIX overlap during larval development and on a cellular level in cell culture. Interestingly, *trim2* morphants showed a clustering and apoptosis of neural progenitors, which are phenotypic hallmarks that are also observed in Alix KO mice. Therefore, we propose that the interaction of Alix and Trim2 plays a key role in the determination and differentiation of neural progenitors via the modulation of cell proliferation/apoptosis during neurogenesis.

## 1. Introduction

Tripartite motif (TRIM) proteins represent a family with more than 80 members in humans. TRIM proteins are characterized by a similar domain structure consisting of a RING-finger domain, one or two zinc finger domains, so-called B boxes, and a coiled-coil domain. Most TRIM family members have been proposed to function as E3 ubiquitin ligases due the presence of their RING-finger domain. The RING-type E3 ligases operate as scaffolds to recruit both ubiquitin coupled E2 and substrate, thereby facilitating ubiquitination [1,2]. Despite their overall similar structural organization, TRIM proteins regulate a wide variety of cellular processes.

One such ubiquitin ligase, TRIM2, is expressed in the mouse brain, predominantly within the cytoplasm of hippocampal neurons, where it interacts with motor protein myosin V [3]. Moreover, Trim2-deficient mice showed an accumulation of neurofilament light chain (NEFL) in neuronal structures, which causes axonopathy, progressive neurodegeneration, and juvenile onset of tremor and ataxia [4]. The re-expression of TRIM2 prevented neurodegeneration via the UbcH5a-dependent degradation of NEFL [5]. Similarly, cultured hippocampal neurons from mice lacking Trim2 showed a loss of axons and neuronal polarity, whereas the overexpression of TRIM2 induced the specification of multiple axons [6]. In mouse embryonic fibroblasts, p42/p44 MAPK-dependent ubiquitination of cell death-promoting factor Bim (*Bcl-2*-interacting mediator of cell death) by TRIM2 conferred neuroprotection [7]. Consistently, alterations of TRIM2 function have been linked to neural diseases in humans. In this respect, miRNAs (miR-9 and miR-181c) have been described, which are down-regulated by amyloid β in Alzheimer’s disease. In particular, miR-181c was found to suppress Trim2 expression, providing a link to progressive neurodegeneration accompanied by juvenile-onset tremor and ataxia [5,8]. Mutations in the TRIM2 gene have been associated with childhood onset of axonal neuropathy [9]. Recently, Trim2 was identified as a cofactor conferring substrate specificity for the interaction of ubiquitin ligase NEDD4-1 and two ABC transporters involved in cholesterol homeostasis [10]. Together, these findings implicate TRIM2 as a key regulator in differentiated neurons, while its function during neurogenesis and neurodevelopment remains enigmatic.

Our present study strongly suggests that in *Xenopus*, Trim2 associates with Pdcd6ip/Alix (Programmed cell death 6 interacting protein/Apoptosis-linked gene-2 interacting protein X) and regulates neural development. Alix is a multimodular adaptor protein involved in the sorting of cargo proteins of multivesicular bodies for incorporation into vesicles and the endolysosome system [11,12,13,14,15,16]. Alix plays a role in the regulation of apoptosis, cell adhesion, cell division, and cytomorphology [17,18]. The overexpression of Alix can promote apoptosis [19,20] or tumor cell proliferation [21], whereas a truncated form prevents apoptosis [19]. Numerous studies have linked Alix to the modulation of apoptosis in neurons and neurodegenerative diseases [19,20,22,23,24]. The lack of Alix expression at the beginning of neurogenesis induces a transient wave of apoptosis in neural progenitors and results in the development of smaller brains in mice [25].

We could show that Trim2 physically interacts with Alix and is possibly involved in a Trim2–Alix/ESCRT-dependent modulation of early neurogenesis. We observed an overlapping expression of *trim2* and *alix* during *Xenopus* development and in cell culture. At early and late tailbud stages, *alix* and *trim2* are both expressed in the central nervous system. The suppression of *trim2* function led to a reduction in brain size caused by increased apoptosis in the head region, phenocopying Alix KO mice. These findings provide evidence that Alix and Trim2 are involved in the timing of determination and differentiation of neural progenitors by the control of cell proliferation and cell survival during early neural development.

## 2. Materials and Methods

### 2.1. Experimental Model, Microinjections, and Plasmids

Albino *Xenopus laevis* frogs were purchased from Nasco (Ft. Atkinson, WI, USA). The production, rearing, and staging of embryos was performed according to Nieuwkoop and Faber, 1967 [26]. All procedures were performed according to guidelines set by the German animal use and care laws (Tierschutzgesetz) and approved by the German state administration Saxony-Anhalt (Projekt/AZ: 42502-3-600 MLU). *trim2* antisense morpholino (5′-CTTCACTGGCCATCCTAGACCACTG-3′) and *alix* antisense morpholino (5′-GTACCGAGATGAAGGTAGCCATCG-3′) oligonucleotides were designed and purchased from Gene Tools, LLC, USA. For antisense microinjections, 1.9 pmol of *trim2* morpholino and 2.5 pmol of *alix* morpholino together with 250 pg synthetic *egfp* or *ß-gal* RNA as a tracer were injected into one cell of two-cell stage embryos. For epistatic analysis, a low concentration of 1 pmol of each morpholino was used in co-injections. A control morpholino oligonucleotide designed by Gene Tools (Philomath, Oregon, USA) was injected as a control (5-CCTCTTACCTCAGTTACAATTTATA-3′). Embryos were raised until the desired stages and fixed in MEMFA. *Xenopus trim2* (Acc. no.: NM_001092023) and *alix* (Acc. no.: NM_001088401) plasmid clones were purchased from Source Bioscience, (Nottingham, UK).

### 2.2. Whole-Mount In Situ Hybridization

To analyze the spatiotemporal expression of respective marker genes, whole-mount in situ hybridization was performed as described earlier [27]. Antisense RNA probes were transcribed in the presence of digoxigenin and/or fluorescein-labeled UTP (Roche, Mannheim, Germany) from plasmids. Chromogenic reactions were carried out using NBT/BCIP (Roche, Mannheim, Germany). For a more detailed analysis of gene expression, 20 µm thick sections were cut from embryos embedded in gelatine/albumin (Roth, Karlsruhe, Germany) using a microtome (Leica, Nussloch, Germany) and were mounted on glass slides. Antisense probes (cut, transcribe): pCS2 + MT-*trim2*: BamHI, T3; pBluescriptKS-*tubb2b*, BamHI, T3; pCS107-*sox3*, HindIII, T7; pBluescriptKS-*myt1*, ClaI, T7; pGEM-T-*pax6L*, NotI, T7; pBst-SK-*otx2*, NotI, T7; pBluescriptKS + -*bf1*, BamHI, T3; pCMVSport6-*pdcd6ip.L*/*alix*, SalI, T7.

### 2.3. Immunostaining of Xenopus Embryos

To analyze the protein expression, either embryos injected with morpholino or mRNA into one cell of a two-cell stage embryo or non-injected embryos were used. Embryos grown until the desired Nieuwkoop-Faber (NF) stage were fixed and left overnight in Dent’s solution at –20 °C for permeabilization. Next, embryos were washed in a series of decreasing methanol concentration steps, blocked for approximately 6 h in 20% horse serum/PBS and incubated with appropriate antibody (see Immunostaining and Western blotting) at 4 °C overnight. Embryos were washed thoroughly in PBSTB twice, 2 h each and in PBSTBN for 2 h. Then, they were washed thoroughly 3 times in a 5 min wash and a wash step in PBSTB at 4 °C overnight. Embryos were transferred to respective secondary antibody conjugated with either alkaline phosphatase or fluorophores and incubated at 4 °C overnight. This was followed by wash steps as above for 30 min and overnight. The staining of embryos was performed as described for Wmish. In case of fluorescence staining, embryos were washed twice for 5 min and left in PBS buffer for further analysis.

### 2.4. Apoptosis/Proliferation Assay in Xenopus Embryos

Apoptotic cells were detected using the TUNEL assay [28]. TdT (Terminal Deoxynucleotidyl Transferase, 20 u/μl) and Dig-11-dUTP were purchased from Roche. Proliferating cells were identified by the detection of phosphorylated histone H3 (PH3) as described [29]. Rabbit polyclonal anti-phosphohistone H3 (Merck-Millipore, Darmstadt, Germany) was used with a dilution of 1:400. Then, embryos were subjected to plastic sections (5 μm), and the PH3-positive cells were counted on each section.

### 2.5. Motility-Escape Assay in Xenopus Embryos

A modified assay based on Sztal et al., 2016 was used in *Xenopus* embryos to perform motility-chase assay [30]. *Xenopus* late tailbud stage control and morphant embryos were put in a Petri dish with 2% agarose cut out in the center into a 1.5 cm × 1.5 cm square well containing 0.1× MBS. Using a micropipette tip, the sensorimotor response of embryos was observed by poking/touching embryo tails. The response to external stimuli was captured by Zeiss microscope and Motic camera and software.

### 2.6. Cell Culture and Transfections

HEK 293T cells were used for the Western blot analysis. For pull-down assay, approximately 1 × 10^6^ cells were seeded in a 6-well plate and maintained in DMEM supplemented with 10% FCS and 100U/mL penicillin G and streptomycin. All media and sera were purchased from PAA laboratories. For transient transfection, 60% confluent cells were transfected with indicated plasmid DNA polyethyleneimine (3 µg DNA in 1 µL of 5mg PEI/mL) (Sigma-Aldrich, Darmstadt, Germany). After approximately 6 h of incubation with the PEI/DNA complex, medium was replaced by fresh serum containing medium. Cells were harvested 24 h post transfection. The lysates were cooked in loading buffer and loaded on 10% SDS gels for further analysis.

### 2.7. Immunocytochemistry and Western Blot

For immunofluorescence, approximately 1.1 × 10^6^ cells of NIH-3T3 and 1.6 × 10^6^ cells of SH-SY5Y were seeded on glass coverslips for 24 h, fixed in 4% paraformaldehyde, permeabilized with 0.2% Triton X-100 for 5 min, and blocked with 2% BSA for 30 min. Following the incubation with the primary antibodies for 45 min at 37 °C, cells were blocked again for 30 min and incubated with the secondary antibodies (anti-mouse-Alexa 488, anti–rabbit-Alexa 594, 1:200). Cells were washed three times with PBS after each step except after blocking and were finally desalted in water, dehydrated in 100% ethanol, and mounted on superfrost glass slides with Mowiol containing DAPI (1:1000). Antibodies: anti-Trim2, Sigma-Aldrich, SAB4200206, rabbit, IHC 1:400, IF 1:200, WB 1:500; anti-Trim2, Novus Biologicals, Wiesbaden, Germany, NB100-1218, goat, IF 1:200, WB 1:500; anti-Alix (3A9), Santa Cruz Biotechnology, Inc., Dallas, Texas, USA, SC-53538. 

### 2.8. Total RNA Extraction, cDNA Preparation, and Semi-Quantitative RT-PCR

Total RNA was extracted from snap-frozen embryos. Embryos were homogenized in TRIZOL and phase separated using chloroform. The mixture was centrifuged and re-extracted using chloroform. Total RNA was precipitated using isopropanol and re-suspended in RNase free water. A total of 500 ng of RNA was used for cDNA synthesis using Protoscript II RTase (NEB) and random primers following manufacturer’s protocol. Semi-quantitative RT-PCR was performed using intron spanning primer pairs (*trim2*-fwd, 5′-CCCGGACGGTAGTGTTACTG-3′, *trim2*-rev, 5′-GTAGTTGACCTGGGGACCTG-3′). Annealing temperatures were 59 °C for both, 29 cycles respectively. Histone h4 (h4-fwd, 5′-CGGGATAACATTCAGGGTATCACT-3′, h4-rev, 5′-ATCCATGGCGGTAACTGTCTTCCT-3′) was used to control the input mRNA (56 °C, 26 cycles).

### 2.9. GST-Pulldown and LC-MS/MS Mass Spectrometry

*Xenopus trim2* was cloned into pGEX4-T1 vector. Fifty micrograms of bacterially expressed and purified GST or GST-Trim2 was added to 1 mL (1 mg protein) lysate and after 1 h at 4 °C under continuous rotation, 50 μL GSH-Sepharose beads were added to the mixture. After 60 min of incubation, the beads were washed three times with IP lysis buffer and once with 0.1 mM Tris, pH7.4. Proteins were eluted with 2× Laemmli buffer, separated by SDS/PAGE, and analyzed by Western blot. GST-Trim2 as a bait was applied on *Xenopus* embryonic lysates (NF stages 30–36) followed by affinity enriched LC-MS/MS on an LTQ Orbitrap instrument [31]. Three independent biological replicates were analyzed. Proteins detected were evaluated based on the intensities of the MS signals and spectral counts to assess the enrichment of protein interactions with GST-Trim2 relative to GST alone. First, proteins are required to be reproducibly present, which is detected by at least five spectral counts. Second, proteins showing less than 100-fold spectral count enrichment over proteins co-isolated with GST alone are deemed nonspecific and are excluded. Third, the base value for Student t-test with a significance of 10^−5^ is set as standard (Table 1).

### 2.10. Alignment, Phylogeny, Synteny

Fasta sequences for the protein families analyzed were obtained by the Blast tool (http://blast.ncbi.nlm.nih.gov) and aligned using the T-Coffee and Box shade tool (http://tcoffee.vital-it.ch/apps/tcoffee/index.html). A phylogenetic tree of the proteins was generated through maximum-likelihood using one-click mode (http://phylogeny.lirmm.fr/phylo_cgi/index.cgi). The synteny analysis is based on data derived with the help of metazome v. 3.2 (https://metazome.jgi.doe.gov/pz/portal.html). The individual gene sequences and the corresponding information regarding *Xenopus laevis* gene loci were obtained from Xenbase (http://gbrowse.xenbase.org) and depicted accordingly.

### 2.11. Statistical Analysis

A Chi-square test of homogeneity examines the extent to which the occupied variables for a four-field table deviate from an expected, homogeneous distribution. Unpaired Chi-square distribution of independence was used to test the null hypothesis. *p*-values were deduced from the abundance values of the replicates of independent biological samples. The significance of the *p* value was set at *p* < 0.001 and degrees of freedom (df) n-1. Null hypothesis is rejected, if the chi square is greater than the value in the chi-square table with *p* ≤ 0.001 and 1 degree of freedom.

## 3. Results

### 3.1. During Xenopus Development, trim2 Is Expressed in Neural and Non-Neural Ectoderm

**Figure 1:**
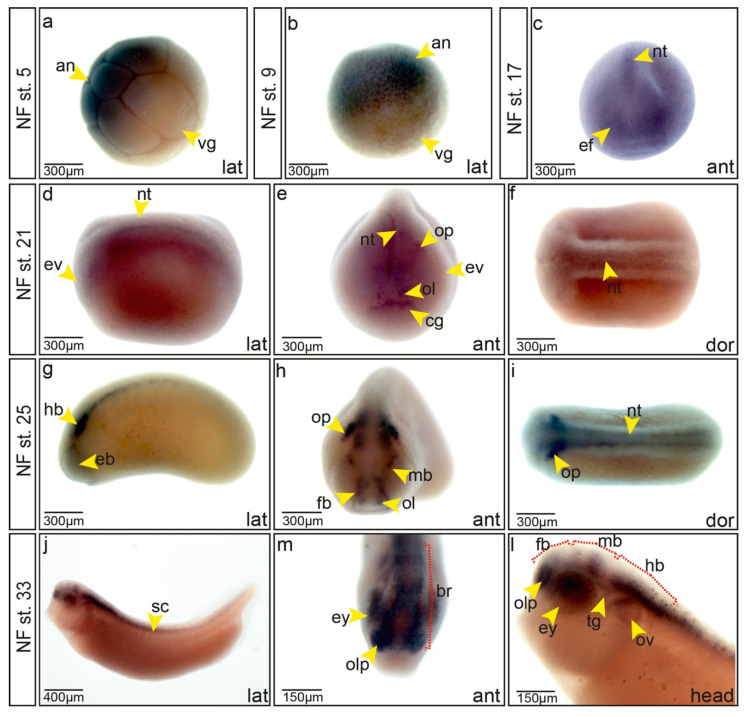
Expression of *trim2* during neurogenesis. (**a**–**i**) *trim2* expression was assessed by whole-mount in situ hybridization (Wmish) at different developmental stages. (**a**,**b**) Maternal *trim2* transcripts were detected in the animal (an) opposed to the vegetal (vg) hemisphere of *Xenopus* embryos. (**c**) NF stage 17, anterior view, *trim2* transcripts were evident in the eye field (ef) and in the forming neural tube (nt). (**d**–**f**) NF stage 21, lateral, anterior and dorsal views. *trim2* transcripts were detected in eye vesicle (ev), neural tube (nt), olfactory (ol), and otic placode (op), as well as cement gland anlage (cg). (**g**–**i**) At NF stage 25, *trim2* expression became more distinct in eye vesicle (eb), forebrain (fb), midbrain (mb), olfactory placode (ol), otic placode (op), and neural tube (nt). (**j**–**l**) NF stage 33, *trim2* in fore- (fb), mid- (mb), and hindbrain (hb), eye (ey), olfactory pit (olp), otic vesicle (ov), spinal cord (sc), and trigeminal ganglion (tg). Scales as indicated. (*strd*-mo *n* = 20 embryos; *trim2*-mo *n* = 16 embryos (E); *p* = 1.97E-09, unpaired χ^2^ test, χ^2^ = 36, df =1, phenotype occurrence (po) =16, *p* < 0.001).

*Trim2* expression was first described in the adult mouse brain [5]. However, its expression during development has not been reported. At first, we examined the temporal expression, performed a sequence analysis, and elucidated the evolutionary relationship of *Xenopus trim2* (Figure 1). Temporal expression analysis showed a gradual decrease of maternal transcripts. First zygotic expression was detected at low levels at the onset of gastrulation and was maintained throughout early development with an increase at the onset of neurulation. A comparison of amino acid sequences revealed that the known domains of TRIM2/Trim2 are fully conserved between mammals and *Xenopus*. Analysis of synteny and phylogeny showed a high conservation of *Trim2*/*trim2* particularly in tetrapods (Figure S1). To analyze the spatial expression of *trim2* during early neural development, we performed a series of whole-mount in situ hybridizations (Wmish) on *Xenopus* embryos. In accordance with the temporal analysis, maternal transcripts of *trim2* were detected in the animal hemisphere of the embryo (Figure 1a,b). At the onset of neurulation, neural plate and forming neural folds showed weak *trim2* expression (Figure 1c). During early neurulation, this expression was more prominent in the prospective head region, where we detected *trim2* transcripts in the neural folds, forming eye vesicles, the prospective brain, olfactory and otic placodes, and the cement gland (Figure 1d–f). At tailbud stages, the expression of *trim2* was detected in the eye, brain, otic vesicle, spinal cord, olfactory pit, and the trigeminal ganglion (Figure 1g–l).

**Figure 2:**
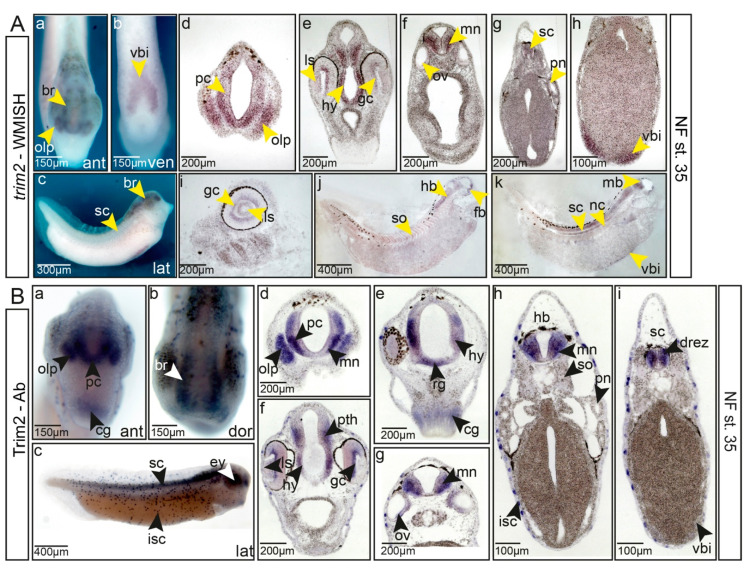
Trim2/*trim2* are expressed in differentiated neurons of the forming central nervous system at the tailbud stage. (**A**) (a–c) Expression of *trim2* mRNA at NF stage 35, anterior, ventral, and lateral views, *trim2* transcripts were detected in the forming brain (br), olfactory pit (olp), spinal cord (sc), and the ventral blood island (vbi). (d–h) Twenty-micrometer gelatine/albumin sections at the level of fore-, mid-, hindbrain, and spinal cord. *trim2* is expressed in the prosencephalon (pc), olfactory pit, ganglion cell layer (gc), lens epithelium (ls), hypothalamus (hy), motor neurons (mn), otic vesicle (ov), and spinal cord (sc). Faint expression was observed in the ventral blood island. (i–k) Sagittal sections reveal *trim2* expression in the ganglion cell layer, lens epithelium, brain, spinal cord, notochord (nc), somites (so), and ventral blood island. (**B**) Whole-mount immunochemistry. (a–c) At NF stage 35 (anterior, dorsal, and lateral views), Trim2 protein expression overlaps strongly with *trim2* mRNA. (d–i) Twenty-micrometer gelatine/albumin transverse sections along the fore-, mid-, hindbrain, and spinal cord region. Trim2 protein was detected in the developing CNS, cement gland, dorsal root entry zone (drez), ganglion cells, hindbrain, hypothalamus, ion-secreting cells (isc), motor neurons, olfactory pit, otic vesicle, prosencephalon (pc), prethalamus (pth), presumptive radial glial cells (rg), somites (so), and ventral blood island.

To investigate whether the timing and appearance of mRNA and protein expression correlate, we compared the whole-mount in situ detection of *trim2* mRNA and immunohistochemical detection of Trim2 protein in late tailbud stage embryos (Figure 2A,B). Histological analysis of sectioned embryos revealed that the temporal and spatial expression of *trim2* transcripts and the Trim2 protein overlap. We observed an elevated expression of *trim2*/Trim2 in the region of prospective motor neurons, crossing fibers of the floor plate, and cells of the medial and lateral ganglionic eminences (Figure 2(Ad–Ag,Bd–Bh)). Lower levels of expression were detected throughout the forebrain (Figure 2(Ad,Ai,Bd)). At the level of diencephalon, Trim2 expression was also observed in the ventral midline region, where radial glial cells are present [32] (Figure 2B). Furthermore, *trim2/*Trim2 was found in the hypothalamus, prethalamus, as well as in ganglion cells of the retina and in the lens epithelium (Figure 2(Ae,Bf)). Double whole-mount in situ with *tubb2b* revealed that *trim2* was expressed at particularly high levels in secondary neurons of the hindbrain, the inner otic vesicle, dorsal root entry zone, and in dorso-lateral sensory neurons of the spinal cord (Figure S2). Generally, *trim2*/Trim2 were highly expressed in committed and differentiated neuronal cells, whereas the expression level in neural progenitors was almost absent. Outside the forming neural system, we observed weak *trim2* expression only in somites, notochord, and the ventral blood island and in a spotted pattern within the skin (Figure 2(Ab,Aj,Ak,Bc,Bh)). The localization of *trim2* transcripts and protein suggest a functional role of Trim2 during the formation of the central nervous system.

### 3.2. trim2 Knockdown Leads to Smaller Head and Affects Cranial Axis Positioning

**Figure 3:**
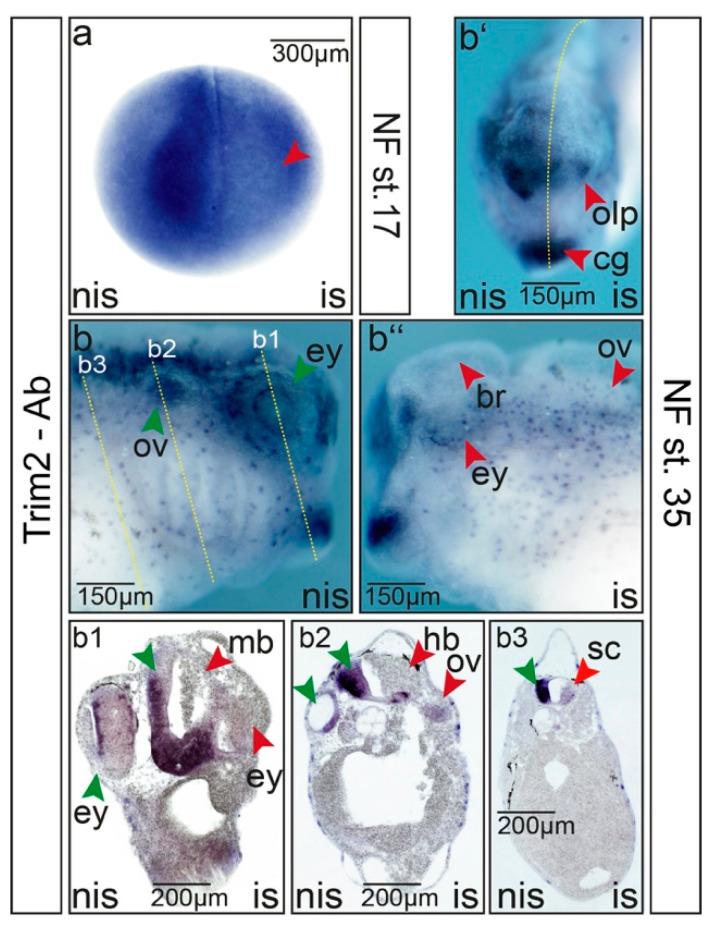
Suppression of *trim2* function interferes with embryonic central nervous system (CNS) development. Microinjections of *trim2*-morpholino were performed into one cell of a two-cell stage embryo. *trim2*-morphants were analyzed by whole-mount immunochemistry using a commercial Trim2 antibody at (**a**) NF stage 17 and (**b**) NF stage 35. (**b**) Non-injected side (nis), (b‘) anterior view, (b‘‘) injected side (is). Forming brain (br), cement gland (cg), eye (ey), olfactory pit (olp), and otic vesicle (ov) showed loss of Trim2 expression. (b1–b3) Twenty-micrometer gelatine/albumin transverse sections of the cranial region. The embryonic brain appeared smaller and disorganized upon *trim2*-morpholino injection. Midbrain (mb), eye (ey), hindbrain (hb), cement gland (cg), otic vesicle, and spinal cord (sc). Red arrowheads represent the affected region of the injected side. Green arrowheads indicate complementary regions of the control side (nis). Scales as indicated.

In order to understand the function of Trim2, we utilized antisense morpholino oligonucleotides to block the translation of *trim2*, while synthetic *egfp*- or *ß-gal*-RNA was co-injected as a tracer into one cell of two-cell stage *Xenopus* embryos. The specificity of the morpholino was confirmed by immunohistochemistry and rescue experiments (Figure 3, Figure S3A) and by injecting a morpholino reporter made of a GFP-tagged complimentary sequence as reported earlier [33]. The immunohistochemistry of *trim2*-morphants at NF stage 17 confirmed the suppression of *trim2* translation within the neural plate territory (Figure 3a). Tadpoles at NF stage 35 showed a strong reduction of Trim2 expression in the eye, brain, and spinal cord upon *trim2*-morpholino injection (Figure 3(b–b’’,b1–b3)) along with a reduction in pigmentation on the injected side (Figure S3B). Moreover, a structural disorganization of presumptive brain tissue toward the non-injected side was also observed (Figure 3b1). Cells were dissociated, misaligned, and clustered all over the presumptive brain and in the forming eye.

### 3.3. Suppression of trim2 Function Affects Neurogenesis, Proliferation, and Cell Survival

**Figure 4:**
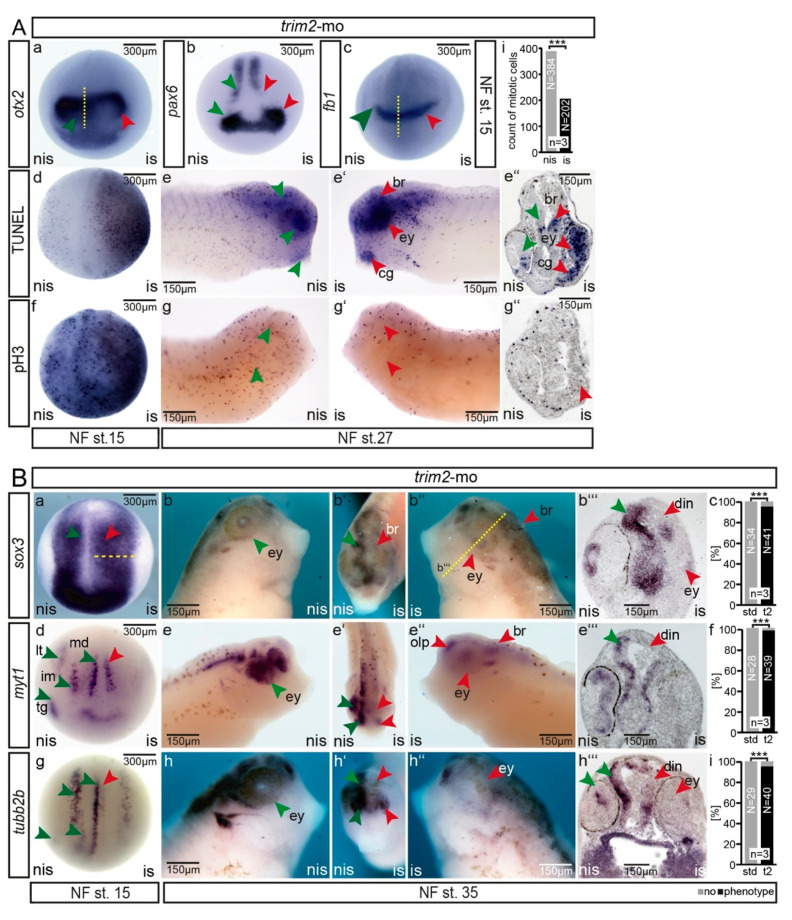
(**A**) Suppression of *trim2* function affected cell proliferation and cell survival within the forming CNS of tadpoles. (a-c; *otx2*: strd-mo *n* = 10E; *trim2*-mo *n* = 12E; *p* = 1.85E-05, unpaired χ^2^ test, χ^2^ =18.33, degrees of freedom (df) =1, po = 11, *p* < 0.001; *fb1*: strd-mo *n* = 10E; *trim2*-mo *n* = 12E; *p* = 2.73E-06, χ^2^ =22, df =1, po = 12). At NF stage 15, early forebrain marker gene expression (*otx2*, *pax6*, and *fb1*) appeared apparently expanded (red arrow heads). However, this is accompanied by an increase in apoptotic cells (d, TUNEL), while proliferation (f, pH3) was not affected at this early stage. In young tadpoles (NF stage 27), the rate of apoptosis was highly increased in the brain region including the forming eye (e’,e’’) and the number of mitotic cells was strongly reduced (g–g’’) (strd-mo *n* = 12E; *trim2*-mo *n* = 20E; *p* = 2.1E-06, unpaired χ^2^ test, χ^2^ =22.5, df =1, po = 18, *p* < 0.001). A similar effect was observed for *fb1* and *otx2* genes (Figure 4(Aa,Ac); (i) Bar plot: cranial region counted for pH3 positive cells (*n* = 3 embryos at NF st. 27; *nis* mean *n* = 384 cells/embryo, *is* mean *n* = 202 cell/embryo, *p* = 0.0074, paired t-test, percentage error 5%, *p* > 0.001). (**B**) Suppression of *trim2* function interfered with secondary neurogenesis**.** (a,d,g) At NF stage 15, *sox3* expression appeared expanded, while the specification and differentiation of neuronal fates appeared reduced as monitored by *myt1* and *tubb2b* (red arrow, yellow dashed line strd-mo *n* = 30E; *trim2*-mo *n* = 28E; *p* = 1.88E-13, unpaired χ^2^ test, χ^2^ = 54, df =1, po = 27, *p* < 0.001; *myt1*: strd-mo *n* = 30E; *trim2*-mo *n* = 28E; *p* = 1.88E-13, χ^2^ = 54, df =1, po = 27; *tubb2b*: strd-mo *n* = 30E; *trim2*-mo *n* = 29E; *p* = 7.17E-13, unpaired χ^2^ test, χ^2^ =51, df =1, po = 27, *p* < 0.001). (b,c) At NF stage 35, *trim2*-morphants showed reduced *sox3* expression in the dorsal intermediate neurons (din) and the lens epithelium (le). (e–i) Similarly, *myt1* and *tubb2b* expression was almost absent in the morphant brain territory (br), particularly in dorsal intermediate neurons (din), eye (ey), olfactory pit (olp). Red arrowheads indicate the affected regions. Green arrowheads represent the corresponding region on the control side; (b) *sox3*: strd-mo *n* = 34E; *trim2*-mo *n* = 41 embryos; *p* = 2.24E-16, unpaired χ^2^ test, χ^2^ = 67, df =1, po = 39, *p* < 0.001); (e) *myt1*: strd-mo *n* = 28E; *trim2*-mo *n* = 39E; *p* = 2.03E-15, χ^2^ = 63, df = 1, po = 38; (h) *tubb2b*: strd-mo *n* = 29E; *trim2*-mo *n* = 40E; *p* = 4.85E-15, unpaired χ^2^ test, χ^2^ = 61, df =1, po = 38, *p* < 0.001. Scales as indicated. (a’’’,c’’’,e’’’) Twenty-micrometer gelatine/albumin transverse sections.

The suppression of *trim2* function resulted in smaller heads, affecting the developing brain and eyes. To investigate whether early forebrain development was impaired in *trim2* morphants, we investigated the expression of early forebrain markers in those embryos. At stage 15, *otx2* expression in the fore–midbrain region was expanded (Figure 4(Aa)). Similarly, the expression of *pax6* in the prospective eye field (Figure 4(Ab)) and that of *fb1* in the presumptive forebrain were expanded. Altered cell proliferation and/or survival might have contributed to the impaired neuronal phenotype upon the suppression of *trim2*. Thus, we monitored mitotic and apoptotic cells accordingly (Figure 4(Ad–Ah)). TUNEL assay revealed a profound increase in the number of apoptotic cells at early neural (NF stage 15) as well as early organogenesis stages (NF stage 27) within the brain, eye, and cement gland (Figure 4(Ad–Ae’’), red arrow heads), and all other territories of *trim2* gene expression. As observed in the cranial transverse section of an NF stage 27 embryo, we often observed an aggregation of apoptotic cells in the presumptive brain and eye on the injected side (Figure 4(Ae’’)). Cell proliferation assay identified only half the number of mitotic cells on the injected side compared to the non-injected side.

To further characterize *trim2* morphants during early neural differentiation, we monitored the expression of three key markers (Figure 4B). Neural precursor cells express the pan-neural marker *sox3* throughout the neural plate at NF stage 15. In *trim2* morphants, *sox3* expression appeared expanded on the injected side, similarly to *pax6* (Figure 4(Ba)). This result suggested a regular, though expanded induction of primary neural precursor cells in *trim2*-deficient cells. However, neural specification and differentiation markers such as *myt1* and *neural-beta-tubulin (tubb2b)*, respectively, which are expressed in medial, intermediate, and longitudinal stripes and in the trigeminal placode within the neural plate, were clearly reduced upon the suppression of *trim2* function (Figure 4(Bd,Bg)) and at the tailbud stage, the expression of *myt1,* and *tubb2b* was significantly reduced (Figure 4(Be–Bi)). Correspondingly, as observed in transverse sections of *trim2* morphants, secondary neural precursor cells marked by *sox3* expression were almost absent in the dorsal regions of the forebrain and midbrain (Fiure 4(Bb)). In placodal-derived structures such as olfactory, otic, and trigeminal placodes, the expression of *myt1* and *tubb2b* was also reduced. Taken together, our results suggest that the suppression of *trim2* function did not impair primary neural induction; rather, it affected neural differentiation and impaired cell proliferation as well as cell survival, maintaining neural fate during early neurogenesis.

### 3.4. trim2 Morphants Show Loss of Motor Sensory Function

Recently, a human patient with a heterozygous missense mutation in TRIM2 was reported to exhibit a loss of gross motor neuron development that resulted in an early onset of axonal neuropathy, muscle hypotonia, and sarcopenia [9]. *Trim2-*depleted mice displayed tremors with episodes of spontaneous seizures [5]. At late tailbud stages, spinal neurons differentiate and connect to create a system that performs sensorimotor function, which is crucial for survival. This sensorimotor function also includes a response to external stimuli such as touch or poking by escaping with a rapid beating movement. Therefore, we performed motility-escape assays on late tailbud stage embryos to investigate whether *trim2* knockdown affects motor-sensory function. The analysis revealed loss of motor and sensory function in *trim2* morphants injected into one cell at the 2-cell stage (Movie 1). After several attempts of poking/stimulating the embryos on the injected side, embryos moved in circles with one-sided beating against stimulation on the non-injected side, which is indicative of a non-spontaneous response to sensory stimulation. A complete loss of motor sensory function accompanied by severe developmental defects and high mortality was observed upon injection of *trim2*-morpholino into both blastomeres of a two-cell stage embryo (Movie 2). We were able to rescue this phenotype as well as a marker gene expression of primary neurogenesis by injecting synthetic mutated *Xenopus trim2* RNA (Δ*trim2*) resistant to the *trim2*-morpholino (Movie 3).

**Movie 1.** Sensory and motor function assessment in *trim2* knockdown embryos. *trim2* morpholino injected into one blastomere at the two-cell stage. NF stage 36 embryos monitored for motility and sensory defects. Inset: control non-injected embryo. Control non-injected embryos *n* = 100 embryos, *trim2-mo n* = 298 embryos, *p* = *** (2.9E-79), unpaired χ^2^ test.

**Movie 2.** Complete loss of motor-sensory function in *trim2* knockdown embryos. *trim2* morpholino was injected either into a one-cell stage embryo or both blastomeres of a two-cell stage embryo. NF Stage 32 embryos monitored for motor-sensory function. Inset: control non-injected embryo. Control non-injected embryos *n* = 20 embryos, *trim2-mo n* = 12 embryos, *p* = *** (1.54E-08), unpaired χ^2^ test.

**Movie 3.** Phenotypic rescue of *trim2* knockdown embryos. Synthetic *Xenopus trim2* mRNA along with *trim2* morpholino was injected into one blastomere of a two-cell stage embryo. NF stage 36 embryos monitored for rescued motility. Inset: control non-injected embryo. Control non-injected embryos *n* = 50 embryos, *trim2-mo n* = 47 embryos, *p* = *** (4.64E-21), unpaired χ^2^ test.

### 3.5. Trim2 Interacts with Alix Physically

**Figure 5:**
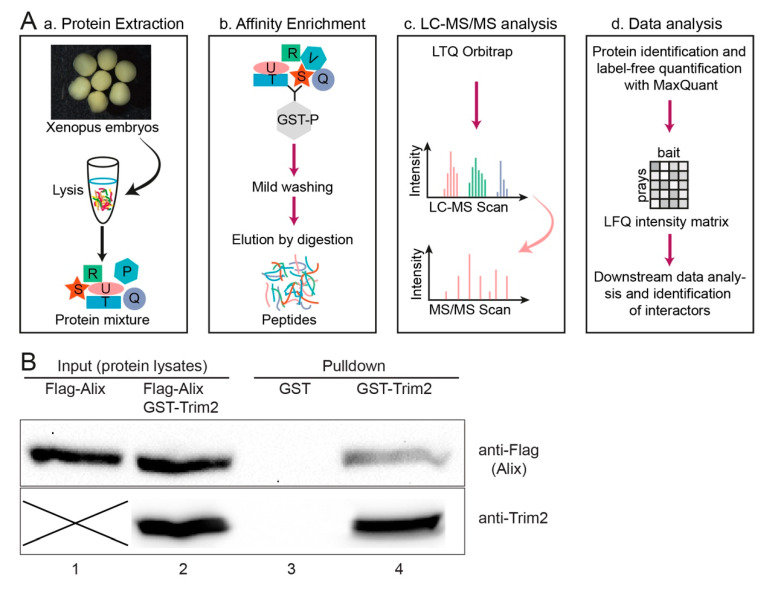
(**A**) Schematic representation of LC-MS/MS analysis for identification of interaction partners, modified after [31]. (a) Protein lysates were extracted from NF stage 30-36 embryos using mild non-denaturing conditions, (b) GST tagged bait protein and protein interactors from the embryo lysates were enriched by GSH-Sepharose. Subsequently, bound proteins were digested into peptides. (c) The peptide mixture was analyzed by single-shot liquid chromatography tandem mass spectrometry (LC-MS/MS) on an Orbitrap instrument (see acknowledgement). (d) Raw data were processed with MaxQuant to identify and quantify proteins and to identify interacting proteins. (**B**) Interaction of Trim2 and Alix was verified by GST pulldown assay using GST-Trim2 as bait and lysates of HEK293T cells transfected with Flag-Alix by Western blot. Cell lysates with input proteins (Lanes 1 and 2). GST alone serves as a control (Lane 3). Eluted flag-tagged Alix after binding GST-Trim2 (Lane 4, upper blot). Note: images of the blots are cropped accordingly to the area of exposed protein bands. Uncropped blots in Figure S4. Two different gels/bolts were used to show the eluted flag-tagged Alix and precipitated GST-Trim2 of the same sample.

Previous studies have shown that NEFL (Neurofilament Light) is a substrate for TRIM2-catalyzed ubiquitination and accumulated in the cerebellum of *Trim2* mutant mice [5]. However, using a GST-Trim2 protein as bait, we were unable to pull down NEFL from *Xenopus* embryonic lysates or from HEK293T cells. To identify proteins that interact with *Xenopus* Trim2, we applied GST pulldown along with high-throughput LC-MS/MS screening (Figure 5A). We identified the Alix protein in very high abundance as a prominent candidate for a Trim2-interacting protein (Table 1). We confirmed the interaction of Trim2 with Alix by a GST pulldown assay using GST-Trim2 and lysates of flag-Alix transfected HEK293T cells (Figure 5B, Figure S4).

To address the intrinsic relationship between Trim2 and Alix, we performed a ubiquitination assay (data not shown). Trim2 and Alix were overexpressed in HEK293 cells in the presence of ubiquitin, but we were unable to see any mono- or polyubiquitylation of Alix (data not shown). Thus, Alix is not a substrate of Trim2.

### 3.6. Expression of Alix Overlaps with trim2 in Xenopus Embryos

**Figure 6:**
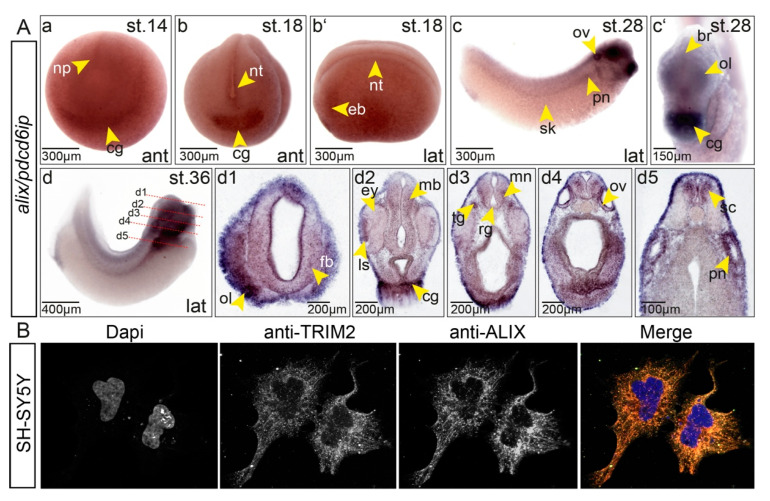
(**A**) Expression of *alix* during neurogenesis overlaps strongly with *trim2* in *Xenopus*. (a) At NF stage 14, *alix* expression was observed within the neural plate (np) and cement gland Anlage (cg). (b–b’, anterior (ant) and lateral (lat) views) At NF stage 18, *alix* transcripts were detected in the neural tube (nt), eye vesicle (ev), and forming cement gland (cg). (c–c‘) At NF stage 28, the presumptive brain (br), olfactory placode (ol), otic vesicle (ov), cement gland (cg), pronephros (pn), and skin cells (sk) reveal *alix* transcripts. (d) At NF stage 36, a lateral view showed strong expression in eye, embryonic brain, spinal cord (sc), otic vesicle, and pronephros. (d1–d5) Twenty-micrometer transverse gelatine/albumin sections taken at the level of fore-, mid-, hindbrain, otic vesicle, and spinal cord. Transcripts of *alix* were detected in the eye (ey), forebrain (fb), midbrain (mb), hindbrain, lens epithelium (ls), motor neuron (mn), olfactory pit (olp), otic vesicles (ov), radial glial cells (rg), spinal cord (sc), and trigeminal nerve (tg). (**B**) Endogenous Alix and Trim2 were detected by fluorescence immunohistochemistry in a strongly overlapping pattern within the cytoplasm of SH-SY5Y glioblastoma cells. Pictures were taken in gray-scale. Merge in false colors. Nuclei were stained with DAPI (blue).

Direct interactions between Trim2 and Alix suggest that both genes are either expressed in overlapping or at least in adjacent domains of the embryo. Indeed, we observed an overlapping pattern of expression for *alix* and *trim2*. During early neurulation, *alix* transcripts were detected in the neural tube, eye vesicle, and the cement gland (Figure 6). At late tailbud stages, high level of *alix* transcripts appeared within the forming CNS and cranial placodes. A high level of expression was further detected in the pronephros, which did not show *trim2* expression (Figure 2(Ag,Bh)), indicating Trim2 independent functions of Alix as well. In addition, we tested by immunofluorescence using commercial antibodies whether endogenous TRIM2 and ALIX do show overlapping expression in a human neuroblastoma cell line SH-SY5Y. Signals for endogenous TRIM2 and ALIX overlapped strongly and showed a filamentous pattern including some vesicular-like or granular-like structures within the cytoplasm (Figure 6B).

### 3.7. Functional Interactions of Trim2 and Alix

**Figure 7:**
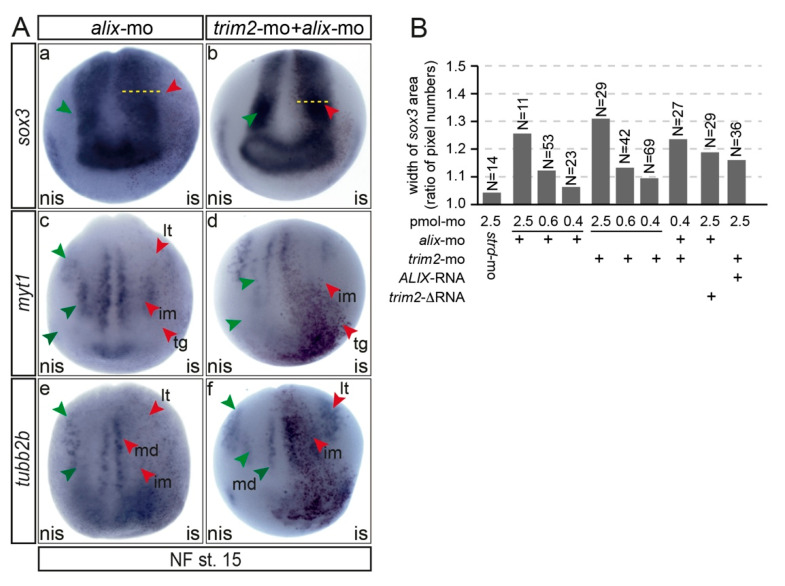
*trim2* and *alix* act in parallel on the formation of primary neurons in *Xenopus*. (**A**) A comparison of early neuronal marker gene (*sox3*, *myt1*, and *tubb2b*) expression at NF stage 15 of *trim2*, *alix,* and combined morphants revealed that primary neurogenesis is likewise impaired. (a–c) An apparent, widened expression of *sox3*-positive neural progenitors in the neural plate region on the injected side (red arrowheads and dotted lines). (d–i) The expression of the neural specification marker *myt1* and the differentiation marker *tubb2b* were repressed or lost upon the suppression of *trim2*, *alix*, and *trim2/alix* function. Primary neurons revealed as medial (md), intermediate (im), longitudinal (lt) stripes, and trigeminal placode (tg) (*myt1* and *tubb2b* positive cells). Figure 7a,b: strd-mo, *n* = 10E; *co-inj. trim2/alix*-mo, *n* = 20E; *p* = 9.47E-06, unpaired χ^2^ test, χ^2^ =19.6, df = 1, po = 17, *p* < 0.001; Figure 7c,d: *myt1*: strd-mo *n* = 10E; *trim2/alix*-mo *n* = 12E; *p* = 1.85E-05, χ^2^ =18, df =1, po = 11; Figure 7c,d: *tubb2b*: strd-mo*, n* = 10E; *trim2/alix*-mo*, n* = 13E; *p* = 1.12E-05, unpaired χ^2^ test, χ^2^ =19, df = 1, po = 12, *p* < 0.001). (**B**) Effect of combined injection of low doses of *trim2* and *alix* mo. To determine the dose of morpholino injection that caused only a very mild phenotype compared to standard morpholino-injected embryos, decreasing amounts of *trim2*-mo or *alix*-mo were injected into one cell of a two-cell stage embryo. The width of the *sox3* expression domain was measured using ImageJ, and the ratio between the non-injected and the injected side was calculated. Combined injection of 0.4 pmol of *trim2* and *alix*-mo each led to an additive effect compared to individual injections (0.4 pmol *trim2*-mo + 0.4 pmol *alix*-mo). The *trim2* morpholino phenotype could not be rescued by co-injection with either h*alix*-RNA (1ng) *n* = 36E, and the *alix*-morpholino phenotype could not be rescued by Δ*trim-2* RNA (1ng) *n* = 29E.

To provide first insight into the functional relationship of Trim2 and Alix, we performed single and co-suppression experiments of *trim2* and *alix* and compared the expression of early neural marker genes. The injection of both morpholinos at a combined dose equal to the final dose of a single *trim2*-morpholino injection resulted in similar alterations of marker gene expression compared to *trim2* morphants (Figure 7). At NF stage 15, *sox3* expression within the neural plate appeared widened on the injected side. The expression of *myt1* and *tubb2b* in intermediate and longitudinal stripes and the trigeminal placode were again reduced. Moreover, at the early tail bud stage (NF stage 32), *alix* morphants showed a similar loss of motor-sensory function as observed for *trim2* morphants (Movie 4). Since Trim2 and Alix physically interacted and combined suppression resulted in an almost identical phenotype, we asked whether an epistatic relationship exists or both act interdependently. To this end, we performed rescue experiments for co-injected morphants with synthetic *trim2*-RNA or human *ALIX*-RNA. However, we were unable to rescue the double morphants with either *trim2* or *ALIX*-mRNA. Further, we tested a dose of *trim2*-mo or *alix*-mo, which alone resulted in only a very mild phenotype. Since the injection of both morpholinos in combination resulted in an additive effect and the *trim2-* and *alix-*morphants could not be rescued by injecting the other RNA, we concluded that Trim2 and Alix interact not linear but in parallel in the same pathway (Figure 7B, Figure S5).

**Movie 4.** Sensory and motor function assessment in *alix* knockdown embryos. *Alix* morpholino injected into one blastomere at the two-cell stage. NF stage 32 embryos monitored for motility and sensory function. Inset: control non-injected embryo. Control non-injected embryos *n* = 30 embryos, *alix*-mo *n* = 45 embryos, *p* = *** (2.9E-79), unpaired χ^2^ test.

## 4. Discussion

Our present work provides insight into the differential expression and function of Trim2 during vertebrate embryogenesis. Trim2 is a conserved member of the TRIM-NHL family of E3 ligases [34]. Evolutionarily, Trim2 is highly conserved across vertebrates (Figure S1). Analysis of *trim2*/Trim2 mRNA and protein expression revealed that *trim2*/Trim2 is predominantly expressed in the developing neural system of *Xenopus laevis* embryos. By the suppression of *trim2* function, we demonstrated that *trim2* affects the proliferation, determination, and differentiation of neural progenitors during embryonic neurogenesis, while primary neural induction was not impaired. Interestingly, swimming tadpoles of *trim2* morphants showed affected motor sensory function at later stages. In addition, we provide evidence that Trim2 physically and functionally interacts with Alix using pulldown techniques, mass spectroscopic analysis, and functional analysis.

In adult mice, *Trim2* expression was reported in regions of the cerebellum, hippocampus, retina, and the spinal cord [5]. In *Xenopus* embryos, we found *trim2* expression in the presumptive ventral brain and spinal cord, including the floor plate, otic vesicles, and olfactory pits. Both Trim2 protein and *trim2* mRNA levels were high in committed and differentiated neuronal cell layers of the future central nervous system. Thus, Trim2*/trim2* expression appeared highly similar in the CNS of *Xenopus* tadpoles and adult mice. In non-neural tissues, only weak expression in head mesenchyme, notochord, somites, and in the ventral blood island was observed.

Recent insights into the mechanisms of neuronal polarization during axon specification revealed TRIM2-dependent post-translational regulation of NEFL [5,6,35]. First reported in the molluscan *Lymnaea stagnalis*, the orthologue L-Trim is up-regulated during the neurite outgrowth of central neurons and in the postnatal brain during neuronal regeneration. A strong inhibition of neurite outgrowth was observed upon the knockdown of *l-trim* mRNA as well [35]. Similarly, cultured hippocampal neurons and motor neurons from mice showed a hyperpolarization-like phenotype upon TRIM2 overexpression and a hypopolarization-like phenotype after *trim2* knockdown. Likewise, proteasomal degradation of NEFL by TRIM2 is necessary for neural polarization of the hippocampal neurons. All data thus far indicate a functional role for TRIM2 in secondary neurogenesis [4,35]. Our findings complement this previous work, providing further evidence of *trim2* function in embryonic neurogenesis. We showed that in *trim2* morphants, the determination and differentiation of proneural genes is impaired at the open neural plate stage, while the precursor cell pool was unaffected or appeared even enlarged. This was also the case as observed for early forebrain markers. Presumably, the suppression of *trim2* function inhibited the cells from exiting the cell cycle properly, thereby resulting in an accumulation of precursor cells with a higher frequency of apoptotic cells. In later stages, we also observed severe gross morphological malformations and a loss of structural organization within the craniofacial region, in contrast to findings in *Trim2^GT^* mice. However, this may be due to the integration of the gene trap vector inside the *Trim2* locus between exons 6 and 7, which may result in a partially active protein, since the catalytically active RING domain of *Trim2* is located on exon 2. In addition, the authors reported a residual expression of nearly 5% of wild-type mRNA due to an alternative splicing effect [5]. On the other hand, the suppression of *trim2* function in *Xenopus* embryos resulted in defective motor neurons as seen by an ataxia-like phenotype of swimming tadpoles, similar to that reported in *Trim2^GT^* mice. 

Little is known about the direct interaction partners of TRIM2/Trim2. We identified Alix (originally described as a pro-apoptotic protein) as a candidate Trim2-interacting protein. First isolated from *Xenopus* oocytes, the *alix* homologue Xp95 is phosphorylated by src kinases [36]. In mice, ALIX has been described as a calcium-dependent interaction partner of apoptosis linked gene-2 (ALG-2) [37,38] and recently of two ABC-type cholesterol transporters of mainly neuronal cellular identity [10]. The overexpression of ALIX in cerebellar neurons of mice revealed activation of the caspase cascade leading to neuronal cell death, independent of the JNK or p38 MAPK signaling pathways. However, a C-terminal fragment of ALIX protected neurons from potassium withdrawal-induced cell death [20]. Our studies suggest a direct, functional interaction of Alix and Trim2 during embryonic neurogenesis and development. However, Alix is not a substrate of Trim2. In general, the expression of *alix* correlated strongly with *trim2* during *Xenopus* development. In tadpoles, both transcripts were found in an overlapping pattern in the developing central nervous system and particularly in the retina and lens, brain, spinal cord, and the olfactory and otic placodes. Moreover, the loss of neural differentiation and the microcephalic-like phenotype observed in *trim2* morphants mimics that of Alix-deficient mice [25]. To date, there is no evidence for a functional role of TRIM2 in the regulation of apoptosis or cell survival. However, there is clear evidence for ALIX in this respect [22,37,38]. Thus, Alix may possess a more general function as an adapter protein, which is important to convey substrate specificity and selection between an E3-ubiquitin ligase such as NEDD4-1 or Trim2 for mono- or polyubiquitination as reported [10,39]. We could show a significant decrease in cellular proliferation, which is most probably due to a high rate of apoptosis within the developing CNS of *trim2* morphants. Along similar lines, a loss of neural progenitors was observed during the transient phase of apoptosis in *Alix* KO mice [25]. ALIX is involved in the regulation of the endolysosomal system through binding to endophilins and to endosomal sorting complexes required for the transport (ESCRT) proteins TSG101 and CHMP4b. Since TRIM2 appears to localize to vesicular-like structures of so-far unknown identity, this may provide a subcellular platform for functional interactions between these referred proteins [11,13,14,15,16,40].

Our results provide evidence for an important function of Trim2–Alix interaction during early neural development. In concert, they may link developmental cues to cellular proliferation/cell survival and thus may take an important function during neuronal determination and differentiation.

## Figures and Tables

**Table 1:** Identified Candidates for Trim2-Interacting Proteins by GST Pulldown and High-Throughput LC-MS/MS.

Gene Name	Gene Symbol	Acc. Number	*p* Value	Abundance
phosphoribosylglycinamide formyltransferase	*gart*	NM_001099882	1.99E-09	99,753,557.71
phosphoglucomutase 3	*pgm3*	NM_001087415	2.27E-08	54,689,274.29
ATP synthase F1 subunit gamma	*atp5f1c*	NM_001091279	4.45E-08	103,066,222.90
serpin family A member 6	*serpina6*	NM_001085634	1.71E-07	6.16
programmed cell death 6-interacting protein	*alix*	NM_001088401	9.27E-07	127,818,404.90
microsomal glutathione S-transferase 3	*mgst3*	NM_001092866	4.76E-06	131.32
ribosomal protein L27a	*rpl27a*	NM_001086720	6.90E-06	10.04
ATP synthase F1 subunit gamma	*atp5f1c*	NM_001087012	8.92E-06	647.69
voltage-dependent anion channel 3	*vdac3*	NM_001091887	1.07E-05	3.32
glyceraldehyde-3-phosphate dehydrogenase	*gapdh*	NM_001087098	1.39E-05	13.55
endoplasmic reticulum oxidoreductase alpha	*ero1a*	BC077754	1.69E-05	19.45
ribonucleotide reductase M1	*rrm1*	NM_001090843	1.93E-05	7.53
CNDP dipeptidase 2	*cndp2*	NM_001093621	2.24E-05	212.52
proteasome 26S subunit, non-ATPase 8	*psmd8*	NM_001091280	5.76E-05	31,524,095.71
heat shock protein family D (Hsp60) member 1	*hspd1*	NM_001086185	7.51E-05	10.39
glutathione S-transferase theta 1	*gstt1*	NM_001091734	1.05E-04	133,086,476.60
adenylosuccinate lyase	*adsl*	NM_001087124	1.20E-04	49,220,858.57
tripartite motif containing 2	*trim2*	XM_018236814	1.66E-04	998,526,258.90
acetyl-CoA acyltransferase 2	*acaa2*	NM_001087263	2.76E-04	21.10
tripartite motif containing 2	*trim2*	NM_001092023	3.28E-04	63.35
aspartyl-tRNA synthetase	*dars*	NM_001092021	3.60E-04	39,632,768.86
ribosomal protein L8	*rpl8*	NM_001086996	5.45E-04	110.75
bifunctional purine biosynthesis protein	*purh*	XM_018238947	6.60E-04	139,875,184.60
cytochrome c-1	*cyc1*	NM_001096171	6.63E-04	13.38
phosphorylase, glycogen; brain	*pygb*	XM_018259948	8.65E-04	43,701,513.71
proteasome 26S subunit, non-ATPase 13	*psmd13*	NM_001094267	1.02E-03	21.91
glutamyl-prolyl-tRNA synthetase	*eprs*	NM_001127874	1.09E-03	22,483,025.14
regulator of chromosome condensation 2	*rcc2*	NM_001095936	1.14E-03	96,777,897.14

List of proteins identified in the interactome from LC-MS/MS analysis. Genes arranged according to decreasing, *p* values. Green shaded lane, Alix protein with one of the highest *p* values and abundance. Note, the identification of Trim2 in the interactome (blue shaded lanes).

## Supplementary Materials

The following are available online at https://www.mdpi.com/2073-4409/9/7/1734/s1, **Figure S1.** (A) *Xenopus* Trim2 protein consists of 748 amino acids and is organized in domains containing a single RING zinc finger, two B-box zinc fingers, a single coiled-coiled, Filamin/ABP 280 domain, and six NHL repeats. (B) Semi-quantitative RT-PCR analysis on consecutive developmental stages. Maternal expression of *trim2* is observed followed by gradual decrease in expression by gastrulation (NF stage 10) and increasing again. Strong expression at neurulation (NF stage 18) until the latest stages analyzed (NF stage 42) in a gradually increasing manner. Expression of *histone H4* was monitored to control total RNA input. (C) Semi-quantitative RT-PCR analysis of *trim2* expression in adult tissues. The brain, eye, and spinal cord showed strong expression, while the skin, heart, kidney, muscle, testis, intestine, and pancreas showed weak expression of *trim2*. (D) Phylogenetic analysis: An evolutionary rooted tree is constructed based on maximum likelihood and bootstrap analysis. The branch length is proportional to the number of substitutions per site. The numbers in red next to nodes represent bootstrap support values. The red arrow indicates the root of the tree, the blue arrow indicates the out-group, and the green arrow indicates the common ancestral node between mammals and amphibians. The bar at the bottom of the phylogram indicates the evolutionary distance to which the branch lengths are scaled based on the estimated divergence. There is a relatively very little divergence observed over time. (E) Synteny organization of *Trim2/trim2*: The chromosomal localization of the gene encoding *trim2* is conserved in mammals. In vertebrates, the locus appeared grossly conserved. Each arrow stands for a single gene. The arrowhead indicates the direction of the ORF. Orthologues are marked with identical colors. *trim2* (black arrow) is present in all species analyzed. Upstream *trim2* is flanked by the same gene with minor exceptions. Downstream *trim2* is flanked by the same gene, except for mammalian. Arrow colors; black: *trim2*, red: *meiotic nuclear divisions 1/mnd1*, yellow: *transmembrane 131 like/tmem131l*, turquoise: *FH2 domain containing 1/fhdc1*, orange: *ADP ribosylation factor interacting protein 1/arfip1*, white 1-10: yet uncharacterized proteins. (F) Comparison of amino acid sequences of TRIM2/Trim2. *H. sapiens* (GenBank Accession no. NP_001123539), *B. taurus* (GenBank Accession no. NP_001077204), *M. musculus* (GenBank Accession no. NP_109631), *R. norvegicus* (GenBank Accession no. NP_001102022), *G. gallus* (GenBank Accession no. NP_001244243), *D. rerio* (GenBank Accession no. NP_001014393), *X. tropicalis* (GenBank Accession no. NP_001005680), *X. laevis (*GenBank Accession no. NP_001085492). Conserved domains are indicated by colored boxes according to A. **Figure S2.** Whole-mount double *in situ* hybridization of *trim2* and *tubb2b* at NF stage 35/36. (a) Lateral view. (b–d) Transverse section of midbrain, hindbrain, and the spinal cord region. *trim2* is mainly expressed in differentiated neurons (e.g., motor neurons, dark blue) such as *tubb2b* (red). **Figure S3.** (A) Phenotypic rescue of *trim2* morpholino-injected embryos. Synthetic *Xenopus trim2* RNA (Δ*trim-2*), which did not contain a complementary sequence of the morpholino, was injected along with *trim2* morpholino into one cell of a two-cell stage embryo. (a) The expanded expression of *sox3*, (c, e) and the medial, intermediate, and longitudinal stripes of *myt1* and *tubb2b-*expressing cells were rescued on the injected side. Red arrows indicate the rescued side. (b, d, f) The percentage of embryos rescued (*sox3 strd*-mo *n* = 20 embryos, *trim2*-mo *n* = 24 embryos, *p* = *** 7.21E-09; *myt1* strd-mo *n* = 18 embryos; *trim2*-mo *n* = 23 embryos; *p* = 6.46E-09; *tubb2b* strd-mo *n* = 20 embryos; *trim2*-mo *n* = 19 embryos; *p* = 1.78E-08, unpaired χ^2^ test). Scale as indicated. (B) Phenotype of *trim2* morphants. (A) Upper panel, whole embryo, lateral views, non-injected side (nis) and injected side (is). Bottom panel shows enlarged cranial lateral view. Red arrowhead indicates a suppression of proper head development (eye, otic vesicle, and branchial arches) and pigmentation. The bar plot represents the survival percentage of *trim2* morphants compared to non-injected control and standard morpholino-injected embryos. Survival rate was 24% for *trim2* morphants. Total injected (ctrl *n* = 186 embryos, *strd*-mo *n* = 288 embryos, *trim2*-mo *n* = 254). Survived (ctrl *n* = 177 embryos, *strd*-mo *n* = 253 embryos, *trim2*-mo *n* = 62). Scales as indicated. **Figure S4.** (A) Uncropped blots of the GST pulldown assay (Figure 5B) at different exposure times. Blot left of red line probed against Alix and the right side against Trim2. The rectangles with dashed lines indicate the presented detail in Figure 5B. The GST-Trim2 lane was loaded as a positive control for the ab reaction only. **Figure S5.** (A) *alix* and *trim2* morphants show an almost identical alteration of marker gene expression. (a–b) Widened expression for *pax6* at the prospective forebrain and eye field region on the injected side in both *trim2* and *alix* morphants was observed. (c–d) forked head box G1 (*fb1*) also displayed a relatively expanded band in *trim2* and *alix* morphants. Yellow dotted line indicates the midline. (B) Phenotypic rescue of *trim2* or alix morpholino-injected embryos. Synthetic *Xenopus trim2* RNA (Δ*trim-2*) or *Human alix* RNA, which did not contain a complementary sequence of the morpholino, were injected along with *trim2* or *alix* morpholino respectively into one cell of a two-cell stage embryo. (a, b) The width of the *sox3* expression domain on the morpholino-injected sides was expanded but (a’, b’) rescued by co-injection of synthetic RNA. The percentage of embryos with no phenotype, phenotype, or rescued phenotype was calculated (*trim2*-mo *n* = 42 embryos, *trim2*-mo plus Δ*trim-2* RNA *n* = 24 embryos, *p* = 3,46E-04; *alix* mo *n* = 52, *alix*-mo plus h*alix*-RNA *n* = 14 embryos, *p* = 2,96E-4).
