# Supplementary material for: Interplay of TRIM2 E3 Ubiquitin Ligase and ALIX/ESCRT Complex: Control of Developmental Plasticity During Early Neurogenesis
Source: Cells. 2020 Jul 20;9(7):1734. doi: 10.3390/cells9071734 (PMC7409263; doi:10.3390/cells9071734)
Supplement: Supplementary file 1 [file cells-09-01734-s001.zip › cells-847924 - Supplementary.docx]

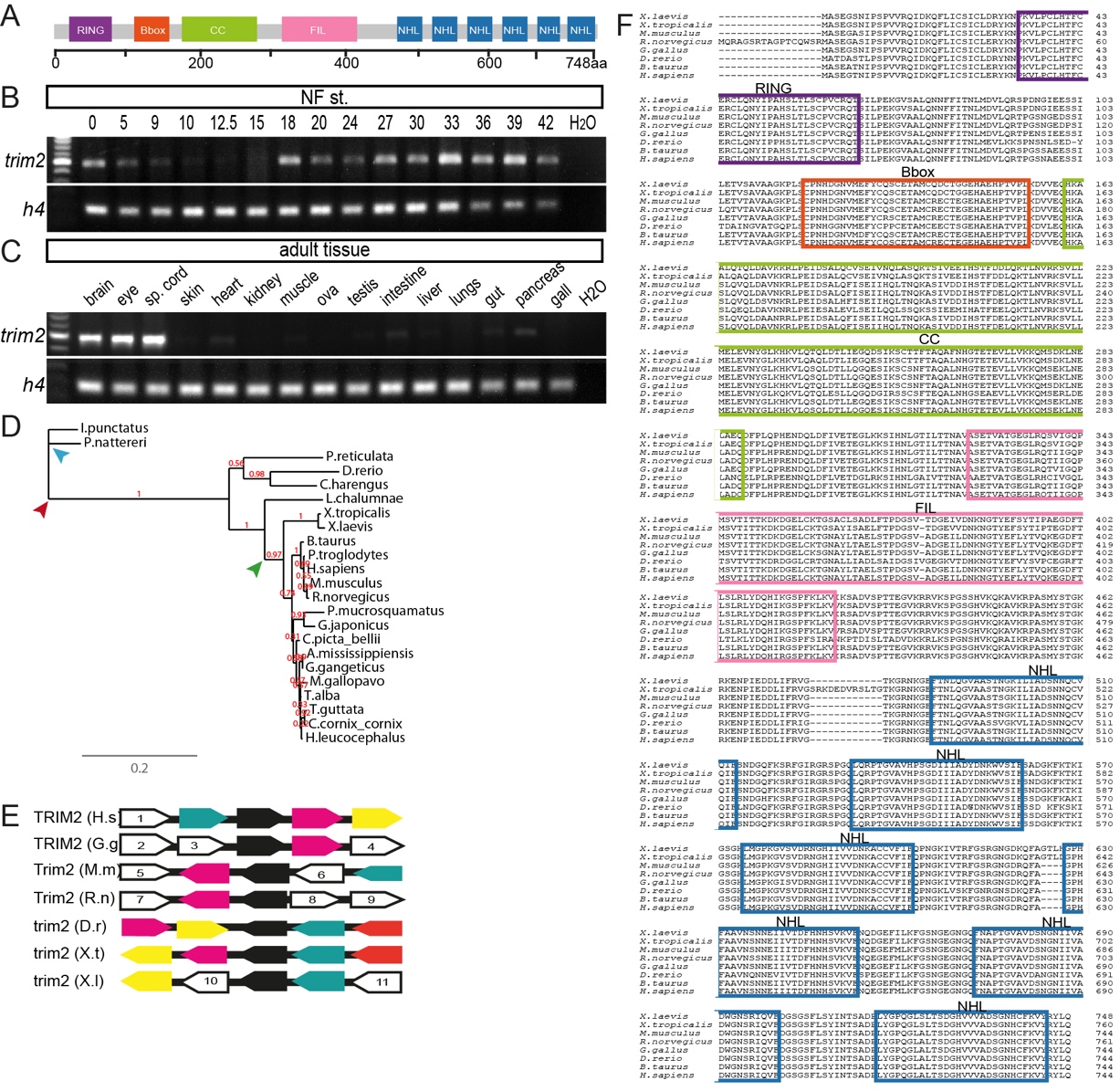


**Figure S1.** (A) *Xenopus* Trim2 protein consists of 748 amino acids and is organized in domains containing a single RING zinc finger, two B-box zinc fingers, a single coiled-coiled, Filamin/ABP 280 domain and six NHL repeats. (B) Semi-quantitative RT-PCR analysis on consecutive developmental stages. Maternal expression of *trim2* is observed followed by gradual decrease in expression by gastrulation (NF stage 10) and increasing again. Strong expression at neurulation (NF stage 18) until the latest stages analyzed (NF stage 42) in a gradually increasing manner. Expression of *histone H4* was monitored to control total RNA input. (C) Semi-quantitative RT-PCR analysis of *trim2* expression in adult tissues. Brain, eye, spinal cord showed strong expression, while skin, heart, kidney, muscle, testis, intestine and pancreas show weak expression of *trim2*. (D) Phylogenetic analysis: An evolutionary rooted tree is constructed based on maximum likelihood and bootstrap analysis. The branch length is proportional to the number of substitutions per site. The numbers in red next to nodes represents bootstrap support values. Red arrow indicates the root of the tree, the blue arrow the out-group and the green arrow indicates the common ancestral node between mammals and amphibians. The bar at the bottom of the phylogram indicates the evolutionary distance, to which the branch lengths are scaled based on the estimated divergence. There is relatively little divergence observed over time. (E) Synteny organization of Trim2*/trim2*: The chromosomal localization of the gene encoding *trim2* is conserved in mammals. In vertebrates the locus appeared grossly conserved. Each arrow stands for a single gene. The arrowhead indicates the direction of the ORF. Orthologues are marked with identical colors. *trim2* (black arrow) is present in all species analyzed. Upstream *trim2* is flanked by the same gene with minor exceptions. Downstream *trim2* is flanked by the same gene, except for mammalian. Arrow colors; black: *trim2*, red: *meiotic nuclear divisions 1/mnd1*, yellow: *transmembrane 131 like/tmem131l*, turquoise: *FH2 domain containing 1/fhdc1*, orange: *ADP ribosylation factor interacting protein 1 /arfip1*, white 1-10: yet uncharacterized proteins. (F) Comparison of amino acid sequences of TRIM2/Trim2. *H. sapiens* (GenBank Accession no. NP_001123539), *B. taurus* (GenBank Accession no. NP_001077204), *M. musculus* (GenBank Accession no. NP_109631), *R. norvegicus* (GenBank Accession no. NP_001102022), *G. gallus* (GenBank Accession no. NP_001244243), *D. rerio* (GenBank Accession no. NP_001014393), *X. tropicalis* (GenBank Accession no. NP_001005680), *X. laevis* *(*GenBank Accession no. NP_001085492). Conserved domains are indicated by colored boxes according to A.


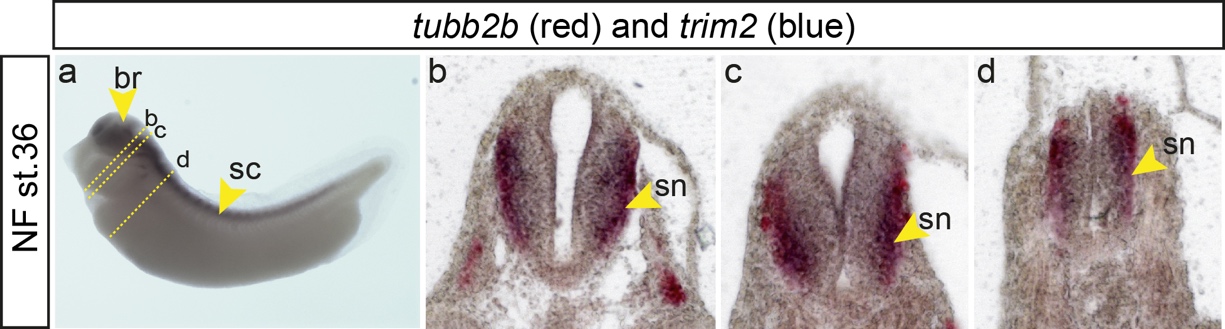


**Figure S2.** Whole-mount double *in situ* hybridization of *trim2* and *tubb2b* at NF stage 35/36. (a) lateral view. (b-d) transverse section of midbrain, hindbrain, and the spinal cord region. *trim2* is mainly expressed in differentiated neurons (e.g. motor neurons, dark blue) like *tubb2b* (red).


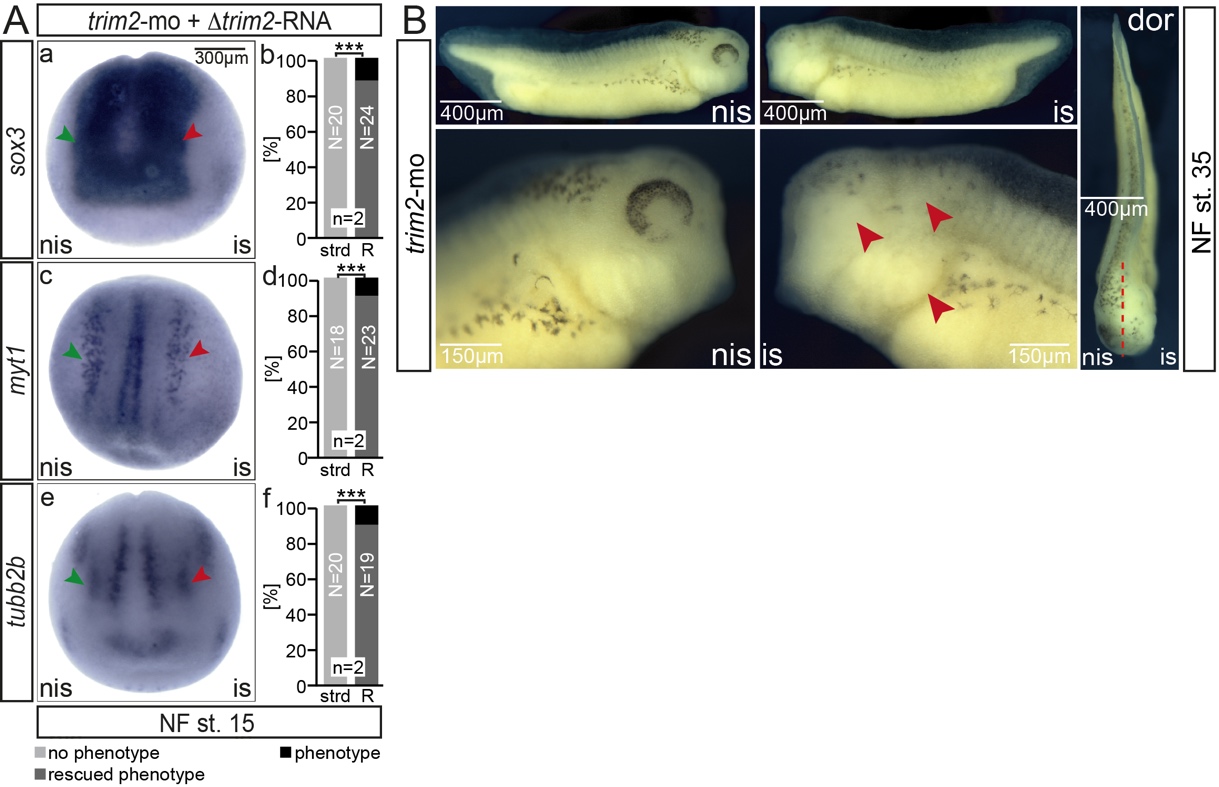


**Figure S3. (A)** Phenotypic rescue of *trim2* morpholino injected embryos. Synthetic *Xenopus* *trim2* RNA (*Δtrim-2*), which did not contain a complementary sequence of the morpholino, was injected along with *trim2* morpholino into one cell of a two-cell stage embryo. (a) The expanded expression of *sox3*, (c, e) and the medial, intermediate and longitudinal stripes of *myt1* and *tubb2b* expressing cells were rescued on the injected side. Red arrows indicate the rescued side. (b, d, f) The percentage of embryos rescued (*sox3* *strd*-mo N=20 embryos, *trim2*-mo N=24 embryos, *p*=*** 7.21E-09; *myt1* strd-mo N=18 embryos; *trim2*-mo N=23 embryos; *p*=6.46E-09; *tubb2b* strd-mo N=20 embryos; *trim2*-mo N=19 embryos; *p*=1.78E-08, unpaired χ^2^ test). Scale as indicated. (B) Phenotype of *trim2* morphants. (A) Upper panel, whole embryo, lateral views, non-injected side (nis) and injected side (is). Bottom panel shows enlarged cranial lateral view. Red arrowhead indicates a suppression of proper head development (eye, otic vesicle and branchial arches) and pigmentation. The bar plot represents the survival percentage of *trim2*-morphants compared to non-injected control and standard morpholino injected embryos. Survival rate was 24% for *trim2*-morphants. Total injected (ctrl N=186 embryos, *strd*-mo N=288 embryos, *trim2*-mo N=254). Survived (ctrl N=177 embryos, *strd*-mo N=253 embryos, *trim2*-mo N=62). Scales as indicated.


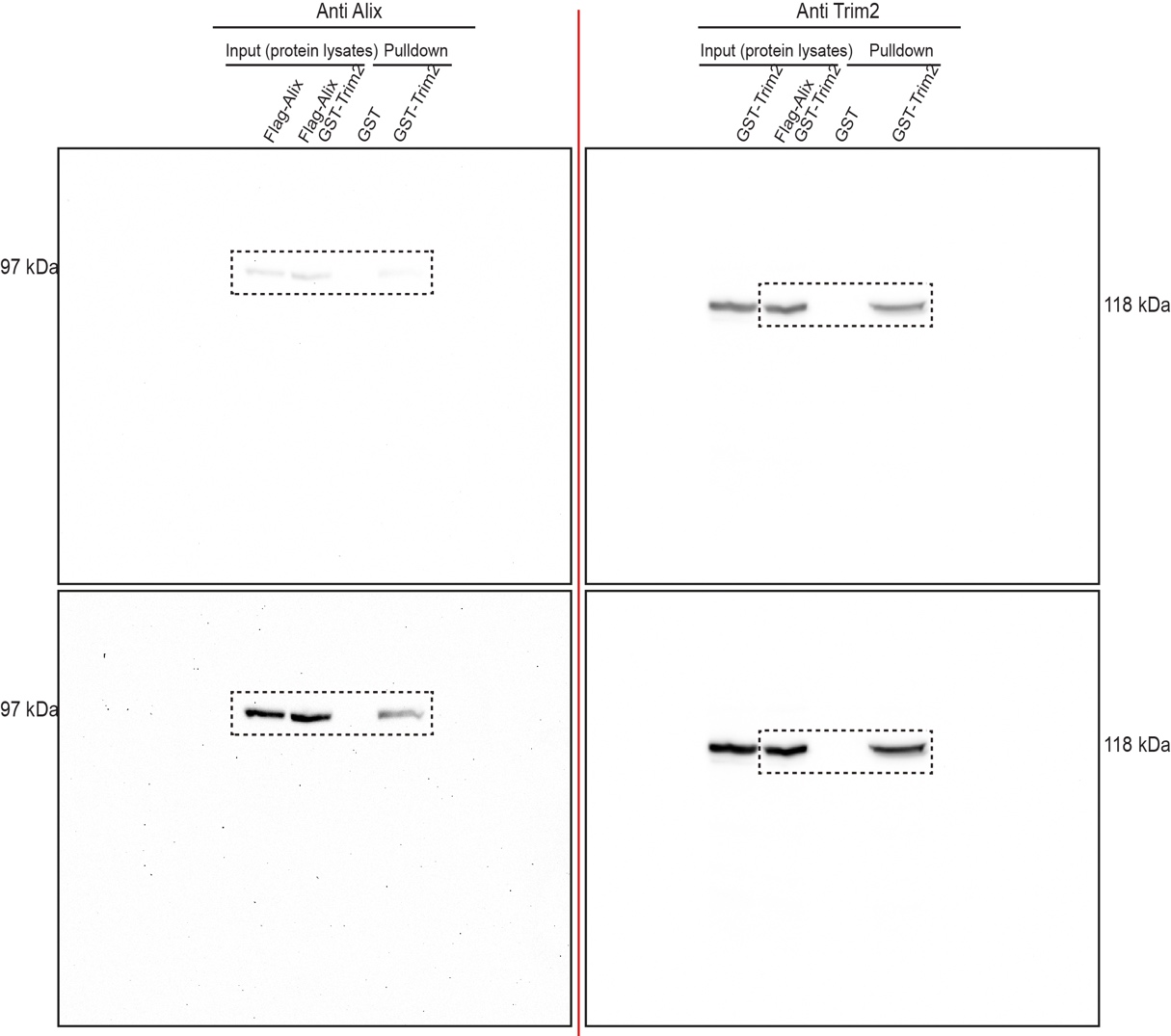


**Figure S4. (A)** Uncropped blots of the GST pull-down assay (Fig.5B) at different exposure times. Blot left of red line probed against Alix and the right side against Trim2. The rectangles with dashed lines indicate the presented details in figure 5B. The GST-Trim2 lane was loaded as a positive control for the ab reaction only.


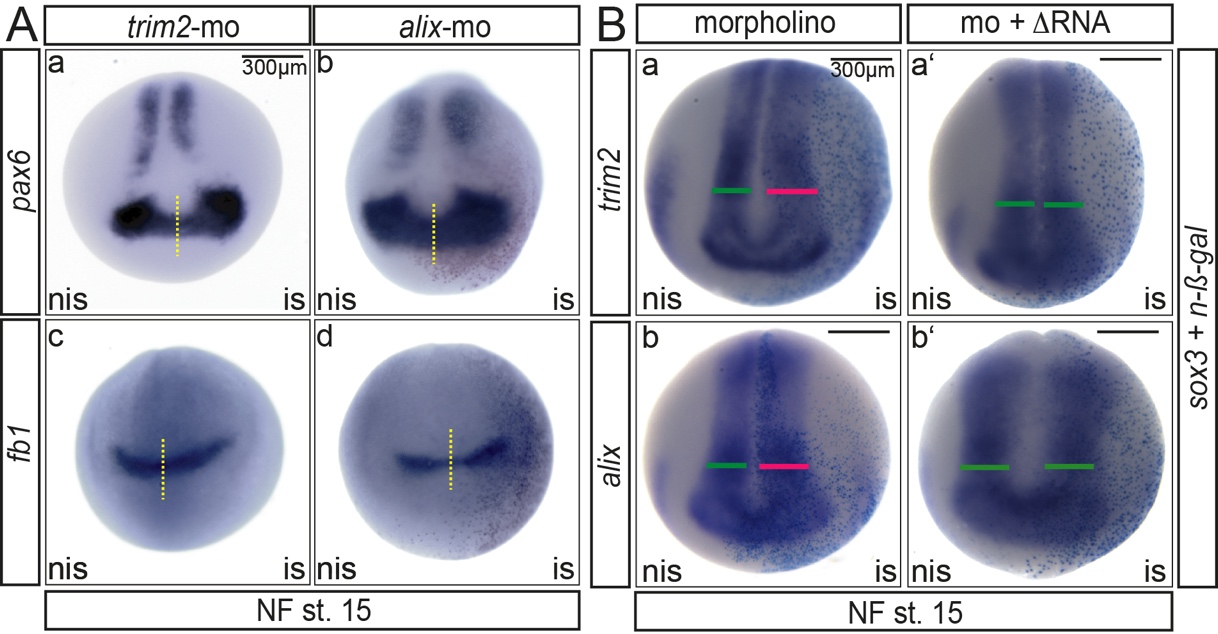


**Figure S5.** (A) *alix* and *trim2*-morphants show an almost identical alteration of marker gene expression. (a-b) Widened expression for *pax6* at the prospective forebrain and eye field region on the injected side in both *trim2* and *alix* morphants was observed. (c-d) forked head box G1 (*fb1*) also displayed a relatively expanded band in *trim2* and *alix* morphants. Yellow dotted line indicates the midline. (B) Phenotypic rescue of *trim2* or *alix* morpholino injected embryos. Synthetic *Xenopus* *trim2* RNA (*Δtrim-2*) or *Human Alix* RNA, which did not contain a complementary sequence of the morpholino, were injected along with *trim2* or *alix* morpholino respectively into one cell of a two-cell stage embryo. (a, b) The width of the *sox3* expression domain on the morpholino injected sides was expanded, but (a’, b’) rescued by co-injection of synthetic RNA. The percentage of embryos with no phenotype, phenotype or rescued phenotype was calculated (*trim2*-mo N=42 embryos, *trim2*-mo plus *Δtrim-2* RNA N=24 embryos, p=3,46E-04; *alix* mo N=52, *alix*-mo plus h*alix*-RNA N=14 embryos, p=2,96E-4).
